# Do Nanoparticles of Calcium Disodium EDTA Minimize the Toxic Effects of Cadmium in Female Rats?

**DOI:** 10.1007/s12011-023-03842-6

**Published:** 2023-09-18

**Authors:** Safa M. Saleh, Osama S. El-Tawil, Manal B. Mahmoud, Sahar S. Abd El-Rahman, Eiman M. El-Saied, Peter A. Noshy

**Affiliations:** 1 Department of Toxicology, Forensic Medicine and Veterinary Regulations, Faculty of Veterinary Medicine, Cairo University, Giza, Egypt; 2 Immune Section, Research Institute for Animal Reproduction, Giza, Egypt; 3 Department of Pathology, Faculty of Veterinary Medicine, Cairo University, Giza, Egypt

**Keywords:** Bone, Chelators, Histopathology, Kidney, Metallothionein, X-ray

## Abstract

The present study aims to investigate the ability of CaNa2EDTA (ethylenediaminetetraacetic acid) macroparticles and nanoparticles to treat cadmium-induced toxicity in female rats and to compare their efficacies. Forty rats were divided into 4 equal groups: control, cadmium, cadmium + CaNa_2_EDTA macroparticles and Cd + CaNa_2_EDTA nanoparticles. Cadmium was added to the drinking water in a concentration of 30 ppm for 10 weeks. CaNa_2_EDTA macroparticles and nanoparticles (50 mg/kg) were intraperitoneally injected during the last 4 weeks of the exposure period. Every two weeks, blood and urine samples were collected for determination of urea, creatinine, metallothionein and cadmium concentrations. At the end of the experiment, the skeleton of rats was examined by X-ray and tissue samples from the kidney and femur bone were collected and subjected to histopathological examination. Exposure to cadmium increased the concentrations of urea and creatinine in the serum and the concentrations of metallothionein and cadmium in serum and urine of rats. A decrease in bone mineralization by X-ray examination in addition to various histopathological alterations in the kidney and femur bone of Cd-intoxicated rats were also observed. Treatment with both CaNa_2_EDTA macroparticles and nanoparticles ameliorated the toxic effects induced by cadmium on the kidney and bone. However, CaNa_2_EDTA nanoparticles showed a superior efficacy compared to the macroparticles and therefore can be used as an effective chelating antidote for treatment of cadmium toxicity.

## Introduction

Cadmium (Cd) is a naturally occurring metal, exists in different oxidational or transitional states. Commercially, Cd is used in television screens, lasers, batteries, paint pigments, cosmetics, and in galvanizing steel and as a barrier in nuclear fission. Cadmium is a toxic non-essential metal that poses a health risk for both humans and animals [1]. Cadmium toxicity occurs from ingestion of contaminated food such as crustaceans, leafy vegetables and rice or water producing long-term health effects. Contamination of drugs and dietary supplements may also be a source of exposure [2, 3]. Cadmium is known to increase oxidative stress by the formation of reactive oxygen species, increasing lipid peroxidation and depleting glutathione and protein-bound sulfhydryl groups. Cadmium also can stimulate the production of inflammatory cytokines and downregulates the protective function of nitric oxide formation [4]. Cadmium causes mutations, DNA strand breaks, chromosomal damage, apoptosis in different organs and impairs DNA repair in cultured mammalian cells [5–7]. Cadmium toxicity is dependent on dose, duration, and route of exposure. It is associated with renal, hepatic, neurological, skeletal, reproductive, and other toxic effects [8, 9].

To ameliorate the toxic effects of Cd, various antioxidants were studied including vitamins such as C and E to reduce Cd-induced oxidative stress. However, Giuseppe et al. [1] stated that caution must be taken with the use of antioxidants in case of Cd toxicity. Long-term intake of high doses of some trace elements, such as zinc, iron, calcium, and selenium may antagonize cadmium toxicity by competitively binding to cadmium binding proteins. However, excessive calcium has many side effects and high doses of zinc leads to impaired immunity. Also, the efficiency of selenium and iron in antagonizing cadmium is limited, as high dose of selenium and iron is poisonous [10–12]. Both in vivo and in vitro studies have demonstrated that some medicinal herbs, containing lupeol, ursolic acid, oleanolic acid, betulinic acid, N-acetylcysteine or carvacrol effectively reduce Cd-induced toxicity via their anti-oxidative, anti-inflammatory, and anti-tumor properties [3, 5, 13, 14]. However, none of the previously mentioned substances can chelate or eliminate residual cadmium or promote its excretion from the body.

Several chelators have been used to treat cadmium toxicity and enhance its excretion; clinically available chelators include ethylenediaminetetraacetic acid (EDTA), British anti-Lewisite (BAL), 2,3-dimercapto-1-propanesulfonic acid (DMPS) and dimercaptosuccinic acid (DMSA). BAL is seldom used clinically because it is more toxic than its derivatives (DMPS and DMSA). Also, cadmium-BAL complex has more nephrotoxic effects than cadmium alone**.** In addition, BAL increases kidney and liver cadmium burdens, may decrease survival, and enhances nephrotoxicity. For these reasons, it is not given in cadmium intoxication [15, 16]. Some studies suggested that EDTA is superior to DMSA in mobilizing intracellular cadmium from the body. Detoxification of cadmium with EDTA has been shown to be therapeutically beneficial in humans and animals when done using established protocols. EDTA was found to decrease Cu toxicity in freshwater fish [17]. Moreover, application of EDTA to the Cd-treated seedlings reduces Cd-induced oxidative injuries by restricting Cd uptake; increasing nonprotein thiols concentration and upregulating most of the components of their antioxidant defense and glyoxalase systems [18]. However, EDTA has side effects on the kidney including renal tubular necrosis and nephrosis when it is used daily with excessive dose, but recovery usually occurs following discontinuation of therapy [7, 19, 20].

Nanotechnology is leading us to a new industrial revolution. The aim of nanotherapy is to drive nanosystems containing recognition elements to act or transport and release drugs exclusively in cells or affected areas to achieve a more effective treatment and minimizing side effects [21, 22]. ZnO and MgO-SiO_2_ nanoparticles were used to mitigate the toxic effects induced by chlorpyrifos and aflatoxins in rats [23, 24]. Also, Karamched et al. [25] found that EDTA-loaded albumin nanoparticles can be used as a chelating agent that reverses arterial calcification in a rat model of chronic kidney disease. In addition, Kataria and Garg [26] demonstrated that EDTA modified Fe_3_O_4_/SC nanocomposites are considered as a promising, low cost and eco-friendly for cadmium removal from the water. Moreover, zinc nanoparticles mitigated the toxic effects induced by silver nanoparticles on the liver, kidney, brain and reproductive system of male rats through counteracting oxidative stress, lipid peroxidation, inflammation, apoptosis and DNA degeneration [27–29]. Nanocurcumin also protected rats against copper oxide nanoparticles-induced hepatorenal toxicity due to its antioxidant, anti-inflammatory and anti-apoptotic properties [30, 31].

To minimize the side effects associated with the use of CaNa_2_EDTA macroparticles for treatment of cadmium toxicity and to optimize the ability to chelate and eliminate cadmium from the body and reduce its residues, the present study was conducted to investigate the efficacy and safety of CaNa_2_EDTa nanoparticles compared to the macroparticles in amelioration of Cd-induced nephrotoxicity in adult female rats.

## Material and Methods

### Animals

Forty female Sprague Dawley rats weighing (150 ± 10 g) were used in this study. They were obtained from the animal house at the faculty of veterinary medicine, Cairo University, Egypt. The animals were kept under observation and acclimatized to the laboratory environment (temperature = 24–26 °C – normal daylight) for one week before the start of the experiment. Rats were maintained in plastic cages (5 rats/cage) and given food and water ad libitum. Animals were examined daily for any clinical abnormality and received humane care in compliance with the guidelines of the national institutes of health (NIH). The institutional animal care and use committee of Cairo University (CU-IACUC) approved the study protocol (approval number: CU-II-20–16).

### Chemicals

**Fig. 1:**
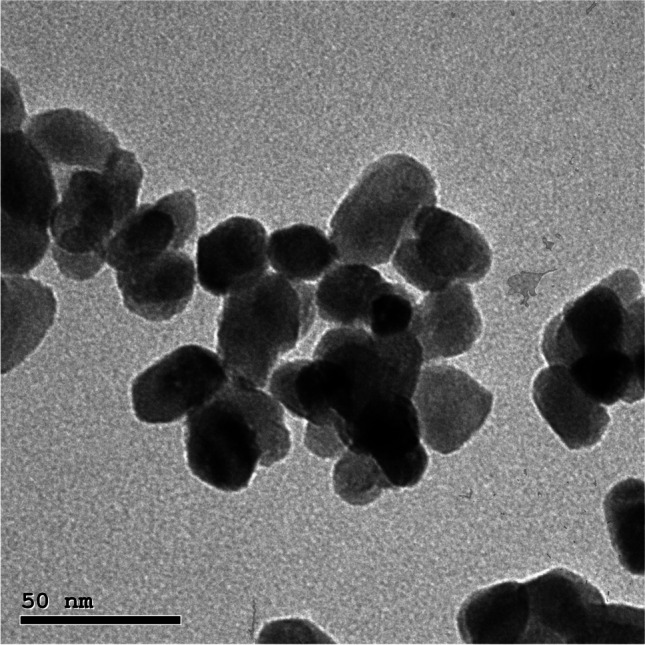
CaNa_2_EDTA nanoparticles (25 ± 5 nm) under transmission electron microscope (TEM)

Calcium disodium EDTA nanoparticles were purchased from Nanotech Co. (Giza, Egypt). CaNa_2_EDTA nanoparticles were in the form of nanospheres of mean size 25 ± 5 nm and were characterized by transmission electron microscopy (TEM) as shown in Fig. 1. Nanoparticles of CaNa_2_EDTA were prepared by nanoprecipitation method and were dissolved in polyvinyl alcohol solution at a definite concentration, and the solution was poured into water-immiscible non-solvent (chloroform) under continuous stirring until a cloudy suspension was formed. Precipitation was formed immediately upon mixing, and then the solution was dried and re-suspended in water [32].

Other chemicals and reagents used in this study were analytically pure and were purchased from Sigma-Aldrich Co. (St. Louis, USA), El-Nasr Co. (Cairo, Egypt) and Kahira Pharmaceuticals and Chemical Industries Co. (Cairo, Egypt).

### Experimental Design

At the start of the experiment, animals were classified into two groups: the control group (10 rats) and the Cd-intoxicated group (30 rats). Cadmium sulfate was added to the drinking water of rats for 10 weeks at a concentration of 60 ppm (mg/L) which provides 30 ppm cadmium. Based on the daily water consumption, the selected concentration was nearly equivalent to ^1^/_20_ of the LD_50_ which is recommended for subchronic toxicity [33]. Lewis [34] and Lide [35] reported that the oral LD_50_ value of cadmium sulfate in rats is 280 mg/kg. In addition, this concentration is less than the concentration of cadmium in some contaminated areas in Egypt [36].

At the end of the 6^th^ week of the experiment, 10 rats from the Cd-intoxicate group were treated with CaNa_2_EDTA macroparticles and another 10 rats from the Cd-intoxicated group were treated with CaNa_2_EDTA nanoparticles. CaNa_2_EDTA macroparticles or nanoparticles were injected intraperitoneally 50 mg/kg/day for four courses (4 days each with an interval of 3 days between the courses) [37].

### Collection of Samples

Every two weeks, rats (10 replicates/group) were weighed, and blood samples were collected from the retro-orbital venous plexus under gentle general anesthesia using ketamine hydrochloride for serum separation. Serum samples were used for measurement of the concentrations of urea, creatinine, cadmium and metallothionein. In addition, 24-h urine samples were collected for estimation of the concentrations of cadmium and metallothionein.

At the end of the experiment (10^th^ week), rats were anesthetized by intra-peritoneal injection of 100 mg/kg ketamine hydrochloride [38] and their skeleton was examined by X-ray and then they were sacrificed for collection of kidney and bone tissue specimens for histopathological examination.

### Kidney Function Tests

Serum urea and creatinine concentrations were determined by using readymade kits obtained from EGY-CHEM Co. (Badr City, Egypt) according to the manufacturer’s instructions.

### Cadmium Concentration

Cadmium concentration was measured in serum and urine by UNICAM 969 Atomic Absorption Spectrophotometer. The samples were prepared according to the method described by Graig and Wayne [39].

### Metallothionein Concentration

Metallothionein (MT) concentration was determined in serum and urine by using Sandwich ELISA kit obtained from Cusabio Co. (Texas, USA) according to the instructions of the manufacturer [40].

### Histopathological Examination

Formalin fixed tissue specimens from kidney and bone were processed for paraffin sections. Sections of about 4–5 μm were stained with Hematoxylin and Eosin (H&E) according to Kieman [41].

### Statistical Analysis

The obtained results were presented as means ± SE. One-way analysis of variance (ANOVA) test was used for comparisons between different groups followed by LSD post-hoc test. The level of significance was set at P ≤ 0.05 using SPSS software (version 16.0).

## Results

### Body Weight

**Fig. 2:**
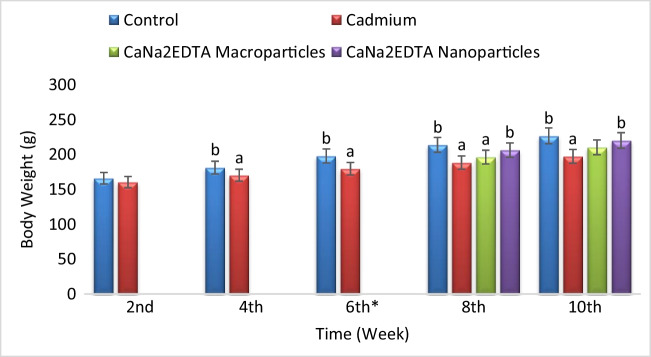
Effects of cadmium, CaNa_2_EDTA macroparticles and nanoparticles on body weight (g) of rats. Values are presented as mean ± SE (*n* = 10 rats/group). ^a^ Significantly different from the control group at *P* ≤ 0.05. ^b^ Significantly different from the Cd-intoxicated group at *P* ≤ 0.05. ^*^ Start of the treatment with CaNa_2_EDTA macroparticles or nanoparticles

Exposure to cadmium resulted in a significant decrease in body weight compared to the control group after 4 weeks of exposure. Treatment of Cd-intoxicated rats with CaNa_2_EDTA macroparticles resulted in a significant decrease in body weight at the 8^th^ week and a non-significant change in body weight at the 10^th^ week compared to the control group and a non-significant change in body weight at the 8^th^ and 10^th^ weeks compared to the Cd-intoxicated group. Treatment of Cd-intoxicated rats with CaNa_2_EDTA nanoparticles resulted in a non-significant change in body weight compared to the control group and a significant increase in body weight compared to the Cd-intoxicated group (Fig. 2).

### Kidney Function Tests

**Fig. 3:**
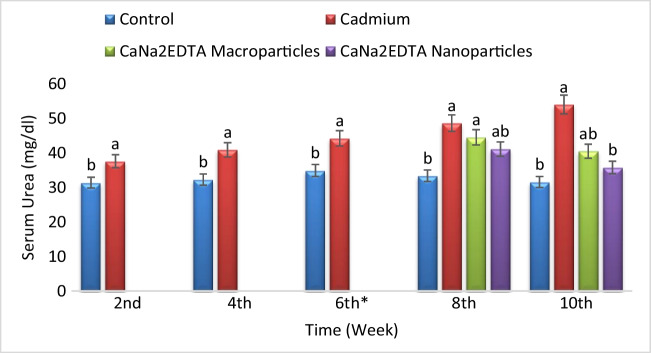
Effects of cadmium, CaNa_2_EDTA macroparticles and nanoparticles on serum urea concentration (mg/dl) of rats. Values are presented as mean ± SE (*n* = 10 rats/group). ^a^ Significantly different from the control group at *P* ≤ 0.05. ^b^ Significantly different from the Cd-intoxicated group at *P* ≤ 0.05. ^*^ Start of the treatment with CaNa_2_EDTA macroparticles or nanoparticles

Exposure to cadmium resulted in a significant increase in serum urea concentration compared to the control group. Treatment of Cd-intoxicated rats with CaNa_2_EDTA macroparticles resulted in a significant increase in serum urea concentration compared to the control group and a non-significant change in serum urea concentration at the 8^th^ week and a significant decrease in serum urea concentration at the 10^th^ week compared to the Cd-intoxicated group. Treatment of Cd-intoxicated rats with CaNa_2_EDTA nanoparticles resulted in a significant increase in serum urea concentration at the 8^th^ week and a non-significant change in serum urea concentration at the 10^th^ week compared to the control group and a significant decrease in serum urea concentration compared to the Cd-intoxicated group at the 8^th^ and 10^th^ weeks (Fig. 3).

**Fig. 4:**
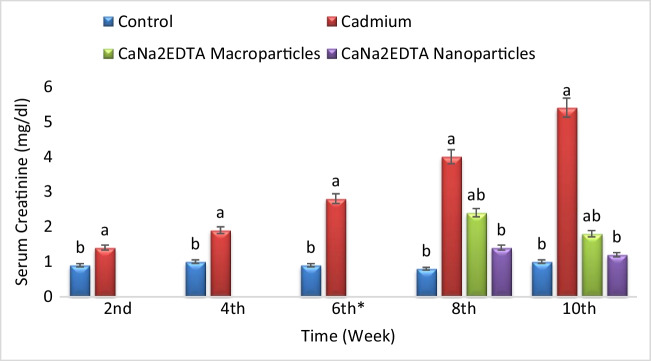
Effects of cadmium, CaNa_2_EDTA macroparticles and nanoparticles on serum creatinine concentration (mg/dl) of rats. Values are presented as mean ± SE (*n* = 10 rats/group). ^a^ Significantly different from the control group at *P* ≤ 0.05. ^b^ Significantly different from the Cd-intoxicated group at *P* ≤ 0.05. ^*^ Start of the treatment with CaNa_2_EDTA macroparticles or nanoparticles

Exposure to cadmium resulted in a significant increase in serum creatinine concentration compared to the control group. Treatment of Cd-intoxicated rats with CaNa_2_EDTA macroparticles resulted in a significant increase in serum creatinine concentration compared to the control group and a significant decrease in serum creatinine concentration compared to the Cd-intoxicated group. Treatment of Cd-intoxicated rats with CaNa_2_EDTA nanoparticles resulted in a non-significant change in serum creatinine concentration compared to the control group and a significant decrease in serum creatinine concentration compared to the Cd-intoxicated group (Fig. 4).

### Metallothionein Concentration

**Fig. 5:**
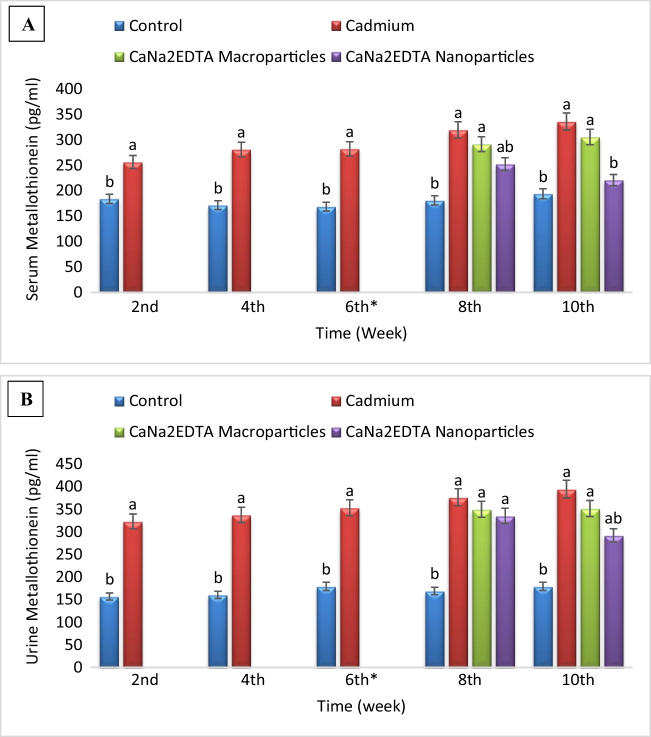
Effects of cadmium, CaNa_2_EDTA macroparticles and nanoparticles on serum (**A**) and urine (**B**) metallothionein concentration (pg/ml) of rats. Values are presented as mean ± SE (*n* = 10 rats/group). ^a^ Significantly different from the control group at *P* ≤ 0.05. ^b^ Significantly different from the Cd-intoxicated group at *P* ≤ 0.05. ^*^ Start of the treatment with CaNa_2_EDTA macroparticles or nanoparticles

Exposure to cadmium resulted in a significant increase in serum metallothionein concentration compared to the control group. Treatment of Cd-intoxicated rats with CaNa_2_EDTA macroparticles resulted in a significant increase in serum metallothionein concentration compared to the control group and a non-significant change in serum metallothionein concentration compared to the Cd-intoxicated group. Treatment of Cd-intoxicated rats with CaNa_2_EDTA nanoparticles resulted in a significant increase in serum metallothionein concentration at the 8^th^ week and a non-significant change in serum metallothionein concentration at the 10^th^ week compared to the control group and a significant decrease in serum metallothionein concentration compared to the Cd-intoxicated group at the 8^th^ and 10^th^ weeks (Fig. 5).

Exposure to cadmium resulted in a significant increase in urine metallothionein concentration compared to the control group. Treatment of Cd-intoxicated rats with CaNa_2_EDTA macroparticles resulted in a significant increase in urine metallothionein concentration compared to the control group and a non-significant change in urine metallothionein concentration compared to the Cd-intoxicated group. Treatment of Cd-intoxicated rats with CaNa_2_EDTA nanoparticles resulted in a significant increase in urine metallothionein concentration compared to the control group and a non-significant change in urine metallothionein concentration at the 8^th^ week and a significant decrease in urine metallothionein concentration at the 10^th^ week compared to the Cd-intoxicated group (Fig. 5).

### Cadmium Concentration

**Fig. 6:**
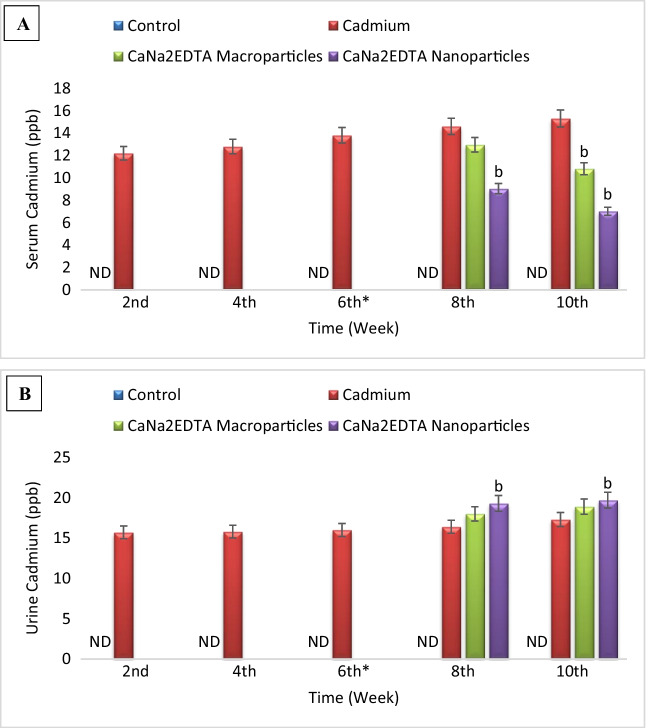
Effects of cadmium, CaNa_2_EDTA macroparticles and nanoparticles on serum (**A**) and urine (**B**) cadmium concentration (ppb) of rats. Values are presented as mean ± SE (*n* = 10 rats/group). ^b^ Significantly different from the Cd-intoxicated group at *P* ≤ 0.05. ^*^ Start of the treatment with CaNa_2_EDTA macroparticles or nanoparticles

Cadmium was not detected in the serum and urine of the control group. Treatment of Cd-intoxicated rats with CaNa_2_EDTA macroparticles resulted in a non-significant change in serum and urine cadmium concentration at the 8^th^ week and a significant decrease in serum cadmium concentration and a non-significant change in urine cadmium concentration at the 10^th^ week compared to the Cd-intoxicated group. Treatment of Cd-intoxicated rats with CaNa_2_EDTA nanoparticles resulted in a significant decrease in serum cadmium concentration and a significant increase in urine cadmium concentration compared to the Cd-intoxicated group at the 8^th^ and 10^th^ weeks (Fig. 6).

### X-ray Examination of Skeleton

**Fig. 7:**
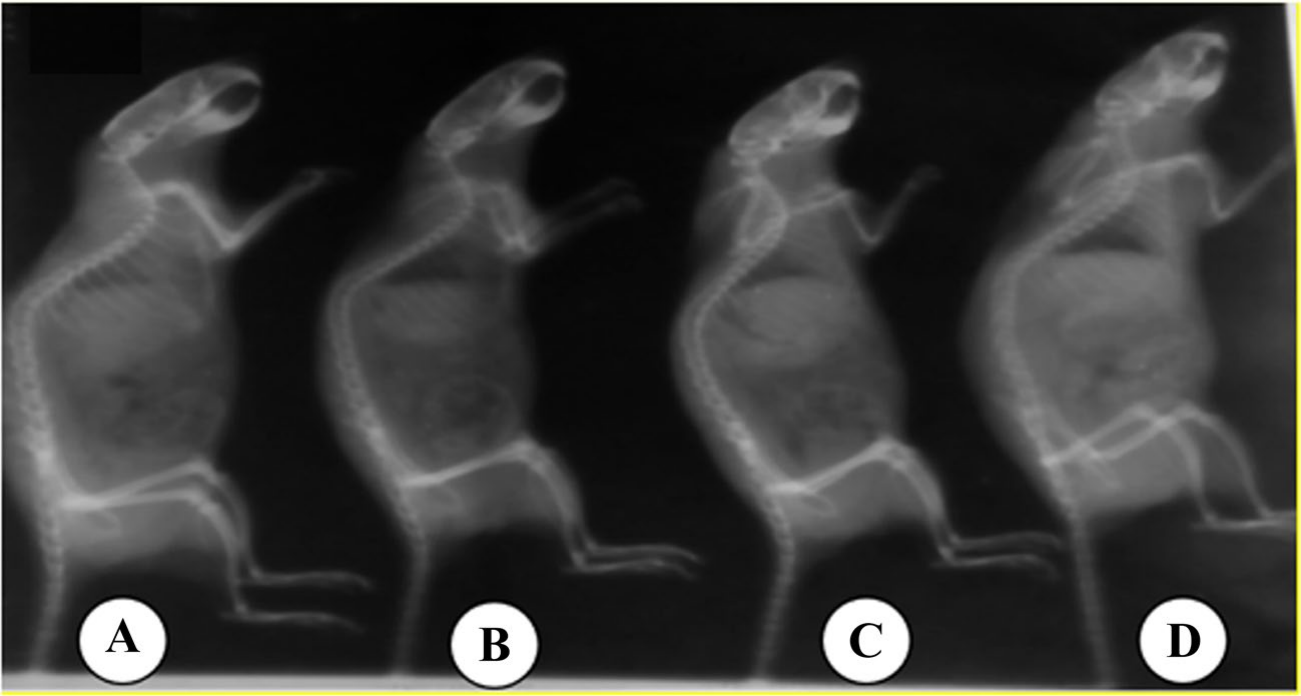
Radiographic lateral view of the skeleton of rats from different groups. **A**: Control rat showing normal radio-density of the skeleton; **B**: Cadmium-intoxicated rat showing clear reduction in bone radio-density of the skull, radius and ulna and metacarpals; **C**: Cadmium-intoxicated rat and treated with CaNa_2_EDTA macroparticles showing moderate reduction in bone radio-density; **D**: Cadmium-intoxicated rat and treated with CaNa_2_EDTA nanoparticles showing mild reduction in bone radio-density. Images were acquired at 36 kV, 100 mA and timed at 0.1 s. The distance from the tube to the image was 70 cm

**Fig. 8:**
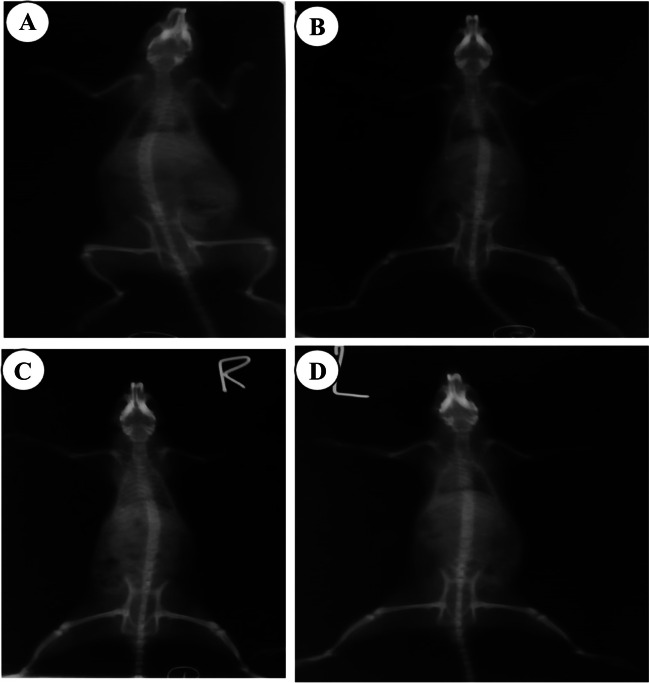
Radiographic ventro-dorsal view of the skeleton of rats from different groups. **A**: Control rat showing normal radio-density of the long bones; **B**: Cadmium-intoxicated rat showing obvious reduction in bone radio-density of the humerus, radius and ulna, tibia and femur; **C**: Cadmium-intoxicated rat and treated with CaNa_2_EDTA macroparticles showing moderate reduction in radio-density of the long bones; **D**: Cadmium-intoxicated rat and treated with CaNa_2_EDTA nanoparticles showing mild reduction in radio-density of the long bones. Images were acquired at 36 kV, 100 mA and timed at 0.1 s. The distance from the tube to the image was 70 cm

The X-ray radiographs of the skeletons of rats from different groups at the end of the experiment are demonstrated in Figs. 7 and 8. The control rat showed a normal radio-density of the skull and long bones (humerus, radius and ulna, femur and tibia). The Cd-intoxicated rat showed a lower radio-density of the skeleton compared to the control rat. The rat intoxicated with cadmium and treated with CaNa_2_EDTA macroparticles showed a lower radio-density of the skeleton compared to the control rat and a higher radio-density of the skeleton compared to the Cd-intoxicated rat. The rat intoxicated with cadmium and treated with CaNa_2_EDTA nanoparticles showed a good radio-density of the skeleton nearly the same as the control rat.

### Histopathological Findings

**Fig. 9:**
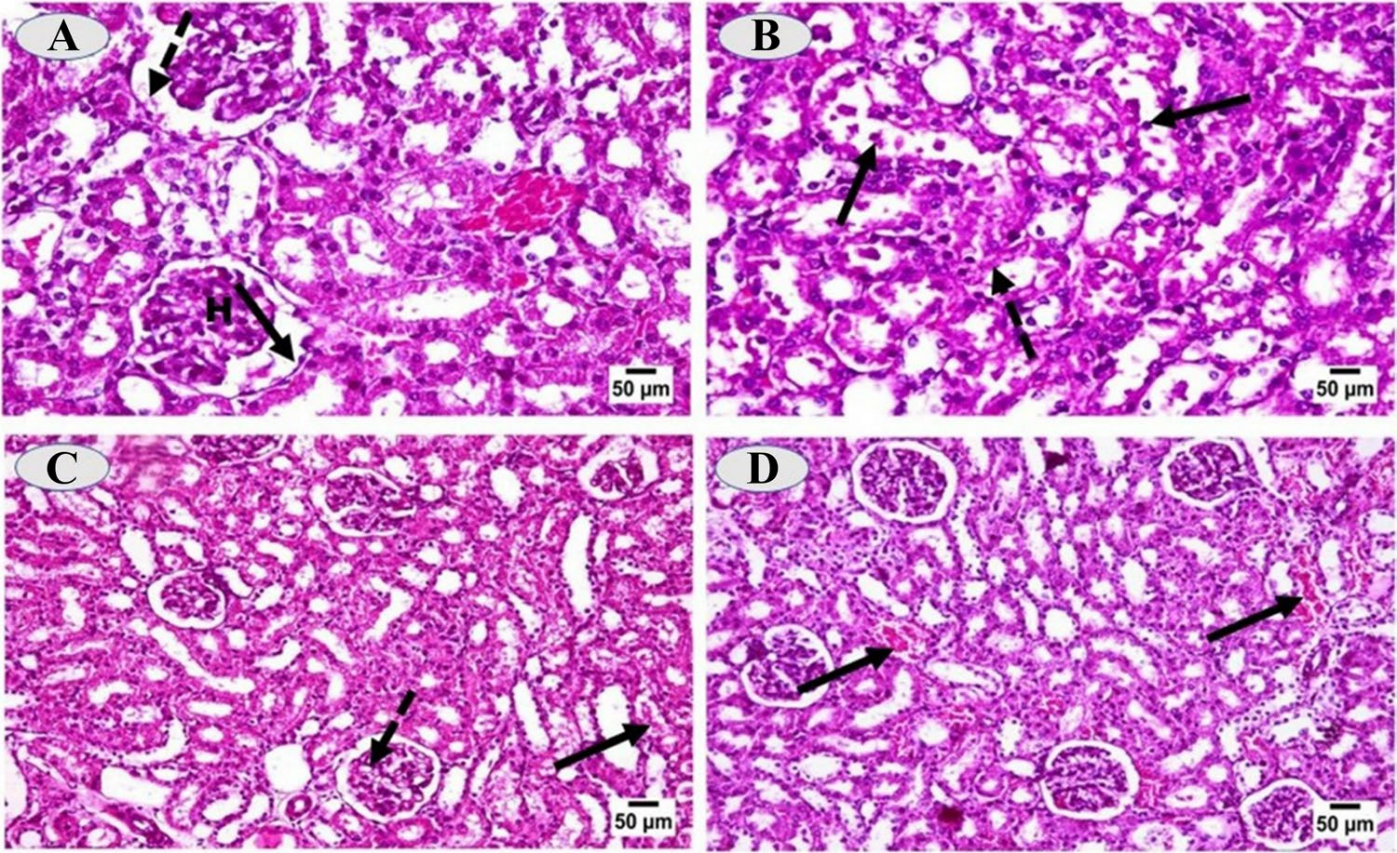
**A**: Kidney of cadmium-intoxicated rat showing hypercellularity of the glomerular tuft (H), thickening of the glomerular basement membrane and the parietal layer of Bowman’s capsule (arrow) as well as presence of granular cast in the Bowman’s space (dashed arrow); **B**: Kidney of cadmium-intoxicated rat showing granular and vacuolar degeneration of the renal tubular epithelium with many necrotic (dashed arrow) and desquamated cells (arrow); **C**: Kidney of cadmium-intoxicated rat and treated with CaNa_2_EDTA macroparticles showing moderate degree of necrotic changes of the tubular epithelial linings, some desquamated cells, granular cast (arrow) and vacuolation of podocytes (dashed arrow) in some the glomerular tufts; **D**: Kidney of cadmium-intoxicated rat and treated with CaNa_2_EDTA nanoparticles showing mild necrotic changes of the renal tubular epithelium, foci of regenerated tubules with restoration of the renal glomeruli and congested interstitial vessels (arrow). (H&E, × 400)

Microscopic examination of tissue prepared sections from kidney and femur of control group revealed normal histological structure. The kidney of cadmium-intoxicated rats showed severe degree of degenerative changes of the renal tubular epithelial linings with many necrotic and desquamated cells (Fig. 9A). Most of the necrotic cells were desquamated in the tubular lumens. The inter-tubular blood vessels were congested. Renal glomeruli were severely affected, most of them showed hypercellularity of their glomerular tufts with thickening of both the glomerular basement membrane and the parietal layer of Bowman’s capsule (Fig. 9B). While kidney of cadmium-administrated rats and treated with CaNa_2_EDTA macroparticles showed mild to moderate degenerative changes of the renal tubular epithelium with scattered necrotic cells, few desquamated cells, and some renal casts in the lumen of some tubules. Some renal glomeruli showed vacuolation of the podocytes (Fig. 9C). Concerning kidney of CaNa_2_EDTA nanoparticles treated rats after cadmium intoxication revealed good restoration of the renal glomeruli with only mild necrotic changes of the renal tubular epithelium and appearance of regenerated foci in the tubules (Fig. 9D).

**Fig. 10:**
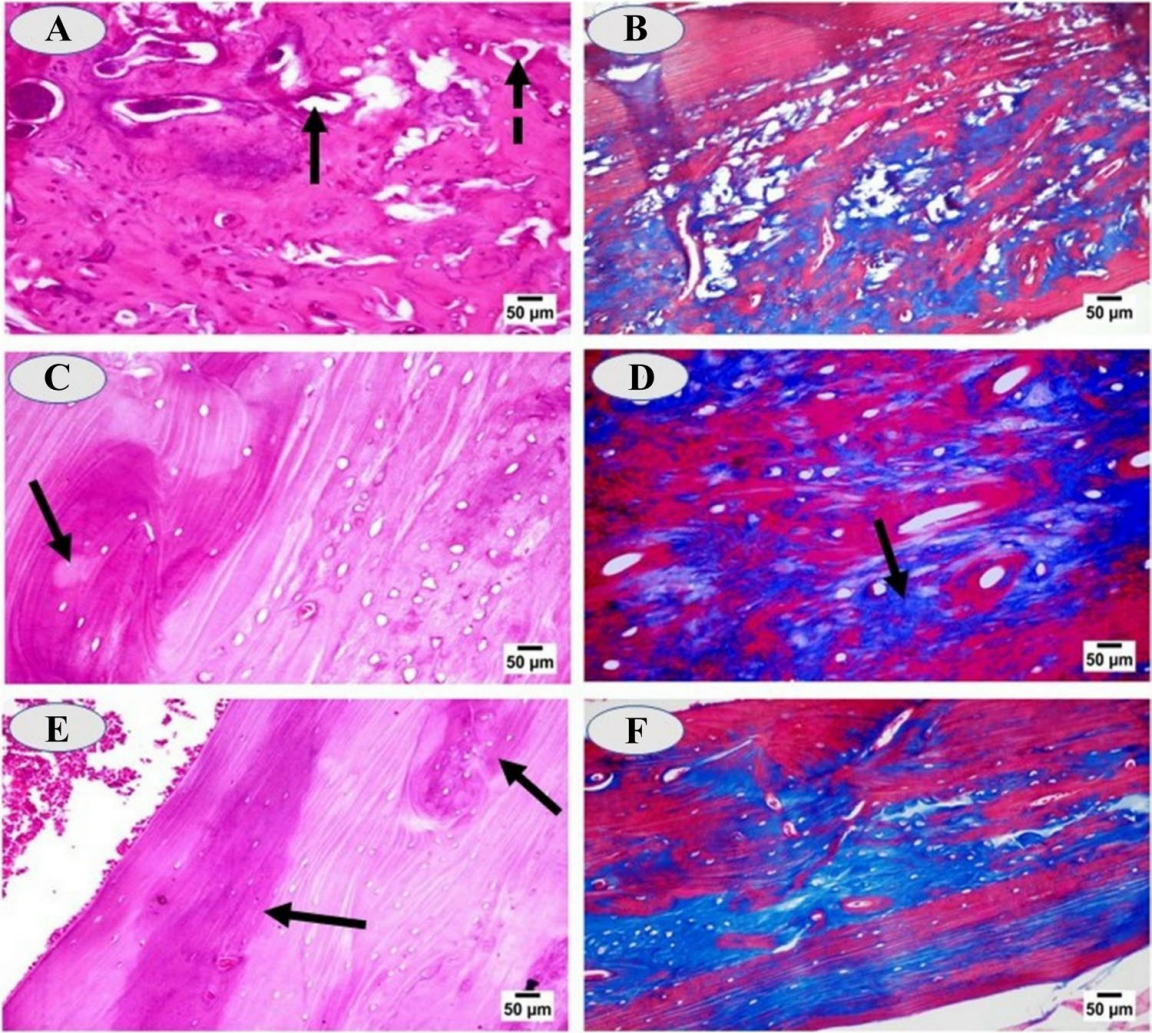
**A**: Cross section of femur diaphysis of cadmium-intoxicated rat showing areas of bone erosions, bony spicules (dashed arrow) and bone resorption (arrow), (H&E, × 400); **B**: Cross section of femur diaphysis of cadmium-intoxicated rat showing areas of bone resorption and decreased collagen fiber staining of bone lamellae, (Masson trichrome stain, × 400); **C**: Section of femur of cadmium-intoxicated rat and treated with CaNa_2_EDTA macroparticles showing small area of bone resorption (arrow) surrounded with dark lines of bone deposition and osteocyte cells inside their lacuna, (H&E, × 400); **D**: Section of femur of cadmium-intoxicated rat and treated with CaNa_2_EDTA macroparticles showing small area of bone resorption with re-deposition of collagen fibers (arrow), (Masson trichrome stain, × 400); **E**: Cross section of femur diaphysis of cadmium-administrated rat and treated with CaNa_2_EDTA nanoparticles showing dark lines of bone deposition (arrow) and osteocytes in their lacunae with regularly and tightly arranged collagen fibers, (H&E, × 400); **F**: Cross section of femur diaphysis of cadmium-administrated rat and treated with CaNa_2_EDTA nanoparticles showing regularly and tightly arranged collagen fibers, (Masson trichrome stain, × 200)

Microscopic examination of femur diaphysis for cadmium-intoxicated rats showed variable-sized areas of bone erosions, resorption, and appearance of bony spicules (Fig. 10A), with decreased density of the collagen fibers. The areas of bone resorption were accompanied with many osteoclast cells inside the resorbed areas; the osteocytes in the vicinity were present in their lacunae (Fig. 10B). Examination of different sections of femur of cadmium intoxicated rats and treated with CaNa_2_EDTA macroparticles showed some areas of bone resorption surrounded with active dark lines of bone deposition (Fig. 10C). Cross section of femur diaphysis of cadmium-administrated rats and treated with CaNa_2_EDTA nanoparticles showed osteocytes inside their lacunae, osteoclast cell inside a small area of bone resorption surrounded with dark lines of bone deposition and redeposition of collagen. Marked dark lines of bone deposition were conspicuously observed with regularly and tightly arranged collagen fibers (Fig. 10D).

## Discussion

Cadmium poisoning is reported in many areas of the world. It is one of the global health problems that affect many organs. Long-term exposure to cadmium through air, water, soil, and food leads to cancer and organ system toxicity such as skeletal, urinary, reproductive, cardiovascular, central, and peripheral nervous, and respiratory systems [16].

In the current study, no mortality was recorded in the experimental groups. This could be due to the low dose used in this experiment (^**1**^/_**20**_ of the oral LD_**50**_). Rats received drinking water contaminated with 30 ppm cadmium showed marked depression and had significantly lower body weight than the control rats. The adverse effect of cadmium on growth performance could be related to alteration of the nutritional absorption and metabolism and reduction of serum proteins production. Tinkov et al. [42] recorded that Cd exposure induces a significant alteration of bacterial populations and their relative abundance in gut, accompanied by increased lipopolysaccharide production, reflecting changed metabolic activity of the intestinal microflora. Gaurav et al. [43], Babaknejad et al. [44] and Jafarpour et al. [45] have reported a similar decrease in body weight.

In addition, cadmium-intoxicated group showed a significant increase in the concentrations of serum urea and creatinine when compared with control group. These increases may be attributed to the toxic effect of cadmium on the renal tubules and glomeruli leading to nephrotoxicity and renal tubular damage.

Cadmium-induced nephrotoxicity is thought to be mediated through cadmium-metallothionein complex, which is synthesized in the liver and released into the blood stream. This complex in the circulation is then filtered through the glomeruli and taken up by the renal proximal tubular cells [12, 46]. Cadmium-intoxicated group showed increased production of metallothionein in serum resulting in an increase of its excretion in the urine. Metallothionein is a cysteine-rich, low-molecular-weight protein. The sulfhydryl group of cysteine can bind cadmium to form a less toxic complex to enhance tolerance of many living organisms including human, animals, snails, and plants to cadmium toxicity [47–51]. In fact, when the hepatic synthesis of metallothionein becomes insufficient for binding all cadmium ions, cadmium not bound to metallothionein produces hepatocyte injury and a cadmium metallothionein complex is released into blood stream. On its way through the kidney, this complex causes injury, mainly in the cortical region, reaching the proximal tubule and causing a gradual loss of the organ’s function [52].

Concentration of cadmium in serum and urine was also significantly increased along the entire period of the experiment. Urinary excretion of Cd is a biomarker of lifetime Cd exposure. Cd excretion in 24-h urine is rather stable in solute composition and is therefore the gold standard to measure Cd in the urinary matrix [3, 53].

In the present work, histopathological examination of the kidney of rats after exposure to cadmium revealed severe degenerative changes of the renal tubular epithelial linings with many necrotic and desquamated cells. Cadmium accumulates in proximal tubule cells and produces a variety of relatively nonspecific toxic effects that result in the death of renal epithelial cells through necrotic or apoptotic mechanisms. Moreover, these histopathological changes could be due to the accumulation of free radicals and increased lipid peroxidation caused by free cadmium ions in the renal tissues [54] and [55]. Numerous studies on cell systems showed that diverse signaling pathways have been involved in cadmium-induced apoptosis [14], but a rise in reactive oxygen species levels, alterations in antioxidant defense system, and stimulation of metallothionein formation are the common phenomena cells follow upon cadmium-induced cytotoxicity.

At the end of the experiment, the X-ray examination of the skeleton of cadmium-intoxicated rats showed low radio-density of the long bones suggesting low minerals deposition or osteoporosis. Microscopic examination of the bone sections confirmed this suggestion as bone erosions and resorption with many osteoclast cells inside the resorbed areas associated with bony spicules and decreased density of the collagen fibers were observed. Several studies on workers exposed to cadmium-polluted fumes and dust showed a connection between cadmium intoxication and bone damage [56]. Cadmium toxicity is associated with the occurrence of Itai-Itai, a disease under which patients show a wide range of symptoms such as low grade of bone mineralization, high rate of fractures, increased rate of osteoporosis, and intense bone-associated pain. Mechanisms of Cd toxicity in bone include stimulation of fibroblast growth factor, which induces phosphaturia and decreases phosphate uptake, leading to osteomalacia [57]. Cd is toxic to MC3T3 osteoblasts [58] and stimulates osteoclasts, thereby inducing osteoporosis [59, 60]. Cd decreases serum osteocalcin levels in rats [61]. These factors apparently combine to induce calciuria, increase bone resorption and decrease bone mineral density in Cd-exposed children [62].

Chelation therapy has been proposed for removing poisonous metals such as Pb, Hg, Cd, and Al. It is considered as a safe and effective strategy to combat metal poisoning [63, 64]. In the present work, treatment with CaNa_2_EDTA macroparticles or nanoparticles offered a pronounced therapeutic effect against sub-chronic cadmium toxicity in female rats consequently less toxicity signs and more improved performance in their body weights were observed. In addition, there was marked improvement in kidney function tests, lower serum cadmium and metallothionein concentrations and higher urine cadmium concentrations when compared to the cadmium-intoxicated rats. Increased urinary cadmium losses by EDTA therapy was also reported by Waters et al. [65]. CaNa_2_EDTA macroparticles or nanoparticles therapy ameliorated the histopathological effects of cadmium on kidney and bone and modulated the skeleton radio-density of intoxicated rats.

All these ameliorative effects were more pronounced in the CaNa_2_EDTA nanoparticles-treated rats when compared with the rats treated with the macroparticles form. These findings suggested that treatment with CaNa_2_EDTA macroparticles or nanoparticles alleviate the toxic effects of cadmium on kidney and bone with special preference to the nanoparticles form. This could be attributed to the more powerful chelating capacity and the higher ability to enhance cadmium excretion of CaNa_2_EDTA nanoparticles and thus most of the cadmium toxic effects were mitigated. The role of CaNa_2_EDTA nanoparticles seems to be due to reduction in size and difference in shape of the nano-formulation, which is evenly spherical because of precipitation process as compared to irregular shape of the micronized forms. The irregular shape of the micronized forms results from machining or grinding processes. The evenly spherical shape of the CaNa_2_EDTA nanoparticles provides an optimization to their use as a chelating agent for cadmium toxicity and thus minimizing its toxic effects [32].

## Conclusion

This study showed the toxic effects of cadmium on the kidneys and bones of rats through biochemical, histopathological, and radiological examinations. Also, the therapeutic effects of CaNa_2_EDTA nanoparticles and macroparticles against cadmium poisoning were demonstrated. It was clear from the results of the present study that CaNa_2_EDTA nanoparticles had a superior efficacy in treating the toxic effects of cadmium on the kidneys and bones when compared to the macroparticles form. Therefore, this study recommends the use of CaNa_2_EDTA nanoparticles (25 ± 5 nm) as an effective chelating antidote to treat cadmium toxicity, enhance its excretion from the body and relieve its signs of toxicity.

## Data Availability

All data generated or analyzed during this study are included in this published article.
